# Does assistive technology contribute to safety among home-dwelling older adults?

**DOI:** 10.1186/s12913-024-11185-8

**Published:** 2024-06-19

**Authors:** Mariya Bikova, Eliva Atieno Ambugo, Trond Tjerbo, Djenana Jalovcic, Oddvar Førland

**Affiliations:** 1 Department of Health and Functioning, Faculty of Health and Social Sciences, Western Norway University of Applied Sciences, Bergen, Norway; 2 Department of Health, Social and Welfare Studies, University of South-Eastern Norway, Vestfold, Norway; 3 Department of Health Management and Health Economics, Institute of Health and Society, University of Oslo, Oslo, Norway; 4 Centre for Care Research, Western Norway University of Applied Sciences, Bergen, Norway

**Keywords:** Home-dwelling older adults, Assistive technology, Safety, Reablement, Dementia care, Long-term care, Norway

## Abstract

**Background:**

Assistive technology carries the promise of alleviating public expenditure on long-term care, while at the same time enabling older adults to live more safely at home for as long as possible. Home-dwelling older people receiving reablement and dementia care at their homes are two important target groups for assistive technology. However, the need for help, the type of help and the progression of their needs differ. These two groups are seldom compared even though they are two large groups of service users in Norway and their care needs constitute considerable costs to Norwegian municipalities. The study explores how assistive technology impacts the feeling of safety among these two groups and their family caregivers.

**Methods:**

Face-to-face, semi-structured interviews lasting between 17 and 61 min were conducted between November 2018 and August 2019 with home-dwelling older adults receiving reablement (*N* = 15) and dementia care (*N* = 10) and the family caregivers (*N* = 9) of these users in seven municipalities in Norway. All interviews were audio-recorded, fully transcribed, thematically coded and inductively analyzed following Clarke and Braun’s principles for thematic analysis.

**Results:**

Service users in both groups felt safe when knowing how to use assistive technology. However, the knowledge of how to use assistive technology was not enough to create a feeling of safety. In fact, for some users, this knowledge was a source of anxiety or frustration, especially when the user had experienced the limitations of the technology. For the service users with dementia, assistive technology was experienced as disturbing when they were unable to understand how to handle it, but at the same time, it also enabled some of them to continue living at home. For reablement users, overreliance on technology could undermine the progress of their functional improvement and thus their independence.

**Conclusion:**

For users in both service groups, assistive technology may promote a sense of safety but has also disadvantages. However, technology alone does not seem to create a sense of safety. Rather, it is the appropriate use of assistive technology within the context of interactions between service users, their family caregivers and the healthcare staff that contributes to the feeling of safety.

**Supplementary Information:**

The online version contains supplementary material available at 10.1186/s12913-024-11185-8.

## Introduction

In Norway, as well as in all industrialized countries, the proportion of people over 65 years and older is growing. By 2060, Norway’s population aged 65 years or above is expected to double [1]. Compared to previous generations, Norway’s population lives longer, is better educated, and has high expectations of a meaningful old age. Norwegian long-term care policy over the last decades has had a strong emphasis on enabling older adults to live at home as long as possible by expanding home-based services [2]. Adding to this, at least in recent years, there has also been a strong policy focus on active ageing, preventive health care, independent living and co-production of care services as a way of enhancing older persons’ quality of life and saving money on public care [2–4]. Nevertheless, the growth in expenditure on long-term care and on homecare has been substantial [5] and perceived as not sustainable by political authorities [6].

Assistive technology (AT) is presumed to have the potential to alleviate costs, compensate for the shortage of care personnel and at the same time support older adults’ quality of life [7–9]. According to the Norwegian governmental plan for the long-term care sector, the so-called ‘Care Plan 2020’, AT can improve users’ ability to manage their own everyday lives, increase the feeling of safety for users and relieve worries for their family caregivers [10].

To date, there is limited consensus on the definition and classification of assistive technologies, and terms like ‘welfare technology’, ‘telehealth’ and ‘telecare’ are often used interchangeably. In this study, we follow the World Health Organization’s definition of assistive technology as “the application of organized knowledge and skills related to assistive products, including systems and services” (11, page 6) and view assistive technology as an umbrella term for assistive products and their related systems and services [11]. Assistive products may be physical products such as wheelchairs, hearing aids, walking sticks, walking frames, alarm buttons and pull cords; or they may be digital and can come in the form of software, sensors and apps to support activities of daily life and communication with care personnel. Assistive products may also be adaptations to the physical environment, such as portable ramps or grab rails installed in different places in a person’s home [11].

In this article we compare two different groups of service users: home-dwelling older people with dementia and people who receive reablement at their homes, and ask how AT impacts their feeling of safety. The groups are chosen because of several reasons: these are two relatively large groups of service users in the Norwegian municipalities; the services they receive constitute considerable costs; both groups are in need of assistance to perform activities of daily living, and both are target groups for assistive technology.

Previous research shows that service users and their family caregivers perceive, experience, and define safety and the feeling of being safe differently. In their integrative review on older people’s perception of safety, Kivimäki et al. described safety as a multidimensional basic need of home-dwelling older adults with positive and negative aspects. Safety includes physical, social, emotional and mental, as well as cognitive safety [12]. AT can be considered as a component of physical safety, whereas home care services and trustful relationships with service providers are within social safety and emotional and mental domains, respectively. Cognitive functioning particularly of older people with dementia is part of cognitive safety that includes acceptance of one’s declining health and awareness of available help.

Both home-dwelling older people with dementia and older people who receive reablement need assistance, but the need for help, the type of help and the progression of their need for help differ; with people with dementia more likely to need more help given that, in addition to facing ageing-related limitations in activities of everyday living, they are also struggling with cognitive problems [13]. Older people receiving reablement, on the other hand, are likely to need less assistance for a half year period after the reablement training [14]. Knowledge about how AT impacts the feeling of safety among these two service groups will yield important information about the possibilities and limitations of AT and may better inform the design of policy and the provision of public services for these groups.

Dementia is a chronic progressive syndrome that causes gradual and irreversible loss of cognitive abilities such as thinking, memory, behavior as well as the ability to perform activities of everyday living; therefore, it entails increased need for support [15]. People who receive reablement, on the other hand, are recovering from health conditions that are not necessarily progressive or irreversible; as such, they are expected to need less assistance to perform activities of everyday living over time. These two groups are seldom directly compared despite the fact that they are two large groups of service users in Norwegian municipalities and constitute considerable costs. This article will contribute with new knowledge regarding similarities and differences in how AT is experienced by such huge, but nevertheless quite different user groups.

Research on service users’ and their family caregivers’ experiences with use and acceptance of AT is growing. However, methodologically, this research often focuses on one service group only and explores either how people with dementia relate to AT [7, 16, 17] or how recipients of reablement do so [18]. In fact, research on reablement users’ use of AT is scarce. To our knowledge, other studies do not distinguish between these two groups and often describe the service users in general terms or simply as older people with various health conditions and in need of assistance. However, AT serves different purposes for these two groups. While for reablement users, the aim is to regain functioning, for people with dementia, the aim is to maintain functioning.

In her realist evaluation of the implementation of telecare, and more particularly, alarm buttons, pull cords and sensors in a medium-sized Norwegian municipality, Berge [7] refers to the service users as “vulnerable” to emphasize their need of increased safety. However, she does not distinguish between the type of services her study participants receive (services for home-dwelling people with dementia, reablement or other services). Hence, even though the study asks for whom, where and when telecare works, by not making a clear distinction between the different types of service users and the differences in the progression of the users’ needs for help and AT, the findings do not allow a comparison of the different service groups. Such a comparison is important for better adaptation of the services for these two groups.

Although there is great optimism regarding the potential of AT to support older adults receiving care services at home, service users increasingly report ambivalence regarding its use [9, 19]. Moreover, different studies show opposing results regarding whether older adults prioritize safety or independence. Robins et al. (2006), for example, found that older adults prioritize safety over independence and that the risk to their safety is a major reason for them to move out of their homes and into institutional settings. A fear of falling, especially when recently discharged form a hospital, is a major concern for home-dwelling older adults [20–22].

Other studies show that service users are more concerned with keeping their independence [23, 24]. The relatives of home-dwelling older adults, however, are more concerned with the safety of their family members especially if the service users had dementia [7, 25]. Berge [7] reported that for older people, the desire to remain living at their homes and the fear of falling were among the major motivating factors for accepting the implementation of AT. The relatives of the older people in Berge’s study felt safer knowing that their family members had AT and could contact the call center should something happen. However, they also reported that their family members preferred receiving home visits rather than relying on technology.

To summarize, research literature does not distinguish between different groups of service users when exploring service users’ and their family caregivers’ experiences with AT. Neither does research literature thematize how the use of AT impacts the feeling of safety among different service groups and the family caregivers.

Hence, this study seeks to answer the question: How does assistive technology impact the feeling of safety among home-dwelling older adults receiving reablement and dementia care, and their family caregivers?

## Methods

### Design and setting

The data analyzed in this article is collected as a part of a larger research project evaluating the ‘Care plan 2020’ [10] for the municipal health and social care services. The research evaluation of the ‘Care plan 2020’ examines how Norwegian municipalities are adapting to demographic changes in the society by looking at the following areas: (1) municipal investments in health and social care services, (2) municipal strategies and innovations for different forms of housing and (3) the effects of services for home-dwelling older adults with dementia and older people receiving reablement [26]. This article is part of component 3, which included 96 in-person interviews in seven Norwegian municipalities. Participants were home-dwelling service users (*N* = 25) and their own family caregivers (*N* = 9), healthcare staff (*N* = 48) and mangers (*N* = 14). For this article, we analyze the interviews with home-dwelling service users (*N* = 25) and their family caregivers (*N* = 9).

The study employed qualitative research design and data was gathered through individual face-to-face interviews with home-dwelling older adults and their own family caregivers. A qualitative design [27] was found appropriate given the study’s focus on an exploration of how assistive technology impacts the feeling of safety among service users and their family caregivers.

### Data collection procedures

#### Inclusion criteria

To ensure that the municipalities reflect the country’s diversity in terms of geography, size and population density, both smaller and bigger, rural and urban municipalities situated in different parts of Norway were selected. The most important selection criteria were that municipalities were taking part in one of the plans in the ‘Care plan 2020’, and had reablement services. We managed to get a diverse sample of seven municipalities that reflect the diversity of the country. Both urban and rural areas were included, as well as smaller and bigger municipalities from different geographical regions. The municipalities are kept anonymous to avoid the possibility of identifying respondents. Leaders of the municipal health and social care services in the seven municipalities suggested healthcare staff, who in turn, suggested participants for the individual interviews. The healthcare staff delivering reablement and homecare services to older people with dementia know the service users well and have the professional competence to assess whether the service users are able to provide informed consent or not. The staff’s expertise and knowledge of the users’ health condition was especially important in the recruitment of service users diagnosed with early stages of dementia to ensure that the participation was voluntary, and that informed consent could be provided.

#### Respondent recruitment

To be included in the study, individual participants had to be home-dwelling older people (age 65+) with early dementia diagnosis and able to provide informed consent. The other group of service users included in the study were home-dwelling older people (age 65+) receiving reablement care. Family caregivers of the respondents were also invited to participate in the interviews. Due to practical reasons such as not living nearby the service user, only few of the family caregivers were able to participate in the interview. Some of the service users in our study did not have family caregivers. Hence the smaller number of family caregivers in our selection.

#### Data collection

Face-to-face, semi-structured interviews were conducted individually with service users (*N* = 25) and their family caregivers (*N* = 9). The interviews were conducted in Norwegian language by the first and fifth authors, between November 2018 and August 2019 at the private homes of the service users. The duration of the interviews was between 17 min and 61 min. The overall theme of the interviews with service users and their family caregivers was their experiences with the homecare services, whether and to what degree they experienced the services as person-centered, well-coordinated and whether the service users could continue living at home with the services they were receiving. The interviews included also questions about safety and whether the provision, implementation and use of AT increased the feeling of safety. For the analysis in this article, we have selected data that focuses particularly on safety regarding the use of and experiences with AT among home-dwelling service users and their family caregivers (Appendix 1 includes the complete interview guide used in component 3 of the research evaluation of ‘Care Plan 2020’).

Characteristics of study participants are presented in Table 1.

**Table 1:** Characteristics of older people (receiving reablement services or services for people with dementia) and their family caregivers

Characteristic	Service users (*N* = 25)	Informal caregivers^a^ (*N* = 9)
	%	%
Female (/male)	60.0	44.4
Age group		
55–64 65–74 75–84 85+	4.0 16.0 48.0 32.0	11.1 66.7 22.2 -
Reablement services (/services for people with dementia)	60.0	-

Note: ^a^Spouses/adult children of service users

### Data processing and analysis

The individual interviews with service users and their family caregivers were audio-recorded, transcribed verbatim, thematically coded and analyzed with NVivo 12 software by the first and fifth authors.

#### Thematic analysis

The data were analyzed following Clarke and Braun’s [28] principles for thematic analysis of qualitative data. The first, second and the fifth authors read the transcripts to familiarize themselves with the data. Next, they inductively generated and collated the first set of codes. These initial codes were organized under potential themes and collated within the identified themes. Then the first author reviewed the themes to identify those that were relevant for this study’s objectives. The first author again reviewed all the codes across the relevant themes with the purpose of identifying anew potential themes and subthemes. The second, third and fifth co-authors reviewed and approved the coding. The feeling of safety related to the use of AT emerged as a major issue in most of the interviews. Henceforth, analytic themes reflecting services users’ and their family members’ experiences with AT were developed. The study’s results are thematically organized and described in the sections that follow. Research ethics approval and consent to participate in this study are described under *Declarations*.

## Results

We first describe the types of technologies that the service users in this study are provided with and then introduce how service users and their family caregivers relate to these technologies.

### Types of technologies

The reablement users in our study are provided with AT aimed at enhancing their recovery by making the activities of daily living more accessible. Among the AT provided to them were walking sticks, walking frames, assistive handles installed in the older person’s bathroom or other places in their home, shower chairs, but also personal digital alarms (pendants) to be activated in case of a fall or other emergencies. Many of the same AT were also provided to the service users with dementia, but in addition, the latter are provided with electronic calendars to remind them of the time and date, electronic medicine dispensers, stove guards and GPS. This finding is in line with findings from related studies from Norway [29, 30].

The biggest difference between the two groups when it comes to the types of technologies is that the home-dwelling service users with dementia are equipped with more technologies to remind them to perform the activities of daily living (e.g., to wake up, eat and take medication), to prevent them from getting lost and utterly to help them maintain functioning and continue living safely at home; whereas service uses receiving reablement have more assistive devices to assist their mobility and help regain functioning while recovering safely at home. However, while most of the informants had been provided with several different assistive devices, in both service groups, one of these devices was the pendant. Hence, the majority of the service users in our study did have experiences with using a pendant.

In the sections that follow, we explore service users’ experiences related to the use of these technologies We focus particularly on the feeling of safety that AT creates for the service users and their family caregivers.

### Experiences with use of assistive technology

The service users’ and their family caregivers’ experiences with AT are explored under the topic ‘Sense of safety related to the use of AT’, which has three major themes. The three main themes and their subsequent subthemes are presented in Table 2.

**Table 2:** Sense of safety related to the use of AT

Theme 1: Feel safe with AT	Theme 2: Feel unsafe with AT	Theme 3: Feel safer with AT when a person attends
When received information about how the device works	When aware of and /or experienced the limitations of the device	When having a contact person in addition to assistive device
When they know how to operate and use the device	When aware of how own physical/cognitive limitations prevent an optimal use of AT	When homecare staff attends to the user
When they have tested the device	When unable to handle the device and/or experience it as disturbing	

In what follows, the main three themes with their subsequent sub-themes are illustrated with quotations from the interviews with service users and their family caregivers.

### Theme 1: feel safe with assistive technology

This theme illustrates different scenarios where AT seems to enhance service users’ and their family caregivers’ sense of safety.

When asked whether they feel confident about continuing living at their own homes despite having conditions that require attendance, several of our informants replied positively. This was the case for both service users receiving dementia care and for service users receiving reablement. For both groups the sense of safety was partly connected to the fact that they had been provided with different types of assistive devices, including personal digital alarms (pendants), which they knew how to operate; and that they had experienced getting quick response from the response center when activating the alarm. In addition, having been provided with enough information about the use and functioning of the assistive device seems to give an extra sense of safety to the service users and their family caregivers.

#### Reablement users

A reablement user living alone, shares that he feels confident that he will get help in case of an emergency, because he has the pendant and knows how to use it:

I: What kind of support do you have if you suddenly get sick?

U: I have a pendant. It gives me safety because I can quickly get help when using it.

I: You have tested it already?

U: Yes, I have.

I: You have activated it?

U: Yes, I have. And I do feel very safe living here (having the alarm).

(reablement user 2, municipality 6)

Another reablement user, who had been hospitalized several times, shares that having the alarm makes him feel safe in his own home despite several occasions of serious falls:

U: Last year I fainted four times and this happened four days in a row. While sitting in my rollator, I collapsed and fell.

I: Do you know whom you can contact or what you should do if your condition gets worse?

U: I can push the alarm button and help will come. I feel completely safe with the pendant.

(reablement user 1, municipality 7)

In addition to the pendant, this service user has been provided with a walking frame and a wheelchair that he uses in his everyday life to get the activities of daily life done. Falling seems to be a common situation for several of the informants in our study. Having been provided with an alarm, however, seems to give a sense of safety as another reablement user also reports:

I: What kind of support do you have in case you feel unwell? Or if the healthcare staff has not arrived yet?

U: I have the pendant.

I: You mentioned the pendant, but how safe do you feel living at home when having a pendant? And do you experience that the healthcare staff contribute to you feeling safe at home?

U: Yes, because they have showed me how to use the assistive devices and this includes the pendant as well.

I: So you do feel safe living at home?

U: Yes, I feel safe.

I: This is good to hear. And how have the last months been for you? Has anything new happened?

U: In fact, I did fall at home, but this was before I got the alarm. It was after the fall I got the alarm.

I: What other assistive devices have they equipped you with?

U: I have special handles several places in the house. I have a special chair to sit on when showering. I do not have a tube anymore. And I have a rollator. Sometimes I use a walking stick.

(reablement user 1, municipality 5)

#### Dementia users

Also the service users receiving dementia care report feeling safe living in their homes when being provided with assistive devices such as pendants, which they know how to operate and experienced getting help when they used it. This is what a service user with dementia shares about living alone at her home:

U: I feel very safe when I have this (shows the alarm). It was me who asked for an alarm and now I feel very safe knowing that I have it.

I: And you just need to push the button …?

U: In case I fall, which has happened many times, it starts beeping and then they (the healthcare staff) come in a short time.

(dementia user 1, municipality 5)

The interviews with service users and their family caregivers show that it is often the family caregivers, and especially the family caregivers of persons with dementia, who request AT. This is because the technology gives a sense of control, and hence safety and a relief, to them as well. The daughter of one of the home-dwelling service users with dementia shares that in addition to the pendant, she has required a GPS for her father (family caregiver of dementia user 2, municipality 7). Another family caregiver, a spouse of a home-dwelling person with dementia, shares the following about his wife having the alarm:

C: We have applied for alarm, and I am sure we will get one. It is safety both for her and for me. Yes, it is a safety. Because when I am not at home, I always wonder where I will find her when I come back home from work. Is she visiting somebody, is she sitting in her chair, or has she had an accident and is lying on the floor? I worry a lot about this.

(family caregiver of dementia user 2, municipality 6)

### Theme 2. Feel unsafe with assistive technology

#### Reablement users

Having the knowledge of the functioning and use of assistive technology does not necessarily create a sense of safety for service users. In fact, being aware of the limitations of AT, such as the limited scope of range of the pendant, can bring a sense of anxiety. The discussion between a reablement user and his wife illustrated with a quotation below, shows some of the challenges related to the use of technology. The user reports that he feels safe when he is at home, but seems at the same time dependent on his spouse being around:

U: But what if I don’t reach the device?

C: Well, you have put it on your wrist.

U: Yes, but what if I am outside the house and I need help?

C: Then you have to cry for help.

U: But there is nobody nearby!

C: Well, the neighbors may hear you, if they are at home.

I: If I understand you correctly, you do not feel very safe outside the house, because the alarm only works within the house. But what about when you are inside the house? Do you feel safe?

U: Yes, I do feel safe at home.

C: Yes, he does. And then you have both me and the alarm. As long as we last (caregiver laughs).

U: But what if something happens with me while you are doing the groceries? It may take some time before you are back from the groceries.

C: Well, then you must remember to wear the pendant alarm.

U: Ok, but what if it is not nearby?

C: You have to remember to have it nearby.

U: But what if you haven’t planned to go to the shop and I haven’t put on the alarm?

(reablement user 1 and family caregiver, municipality 1)

The service user has been receiving reablement care after prolonged hospitalization and has been provided with a pendant that he is instructed to wear on his wrist. However, he forgets to wear the alarm, especially when he is at home, and is thus dependent on his wife reminding him wear the pendant or her being around. Later in this interview when asked whether he will be able to continue living at home with the services he receives, the user states that he would not be able to do so without the help from his wife. It seems that technology limits the user’s possibility to explore and use the physical space around his own home due to the technology’s limited scope of functioning.

Awareness of how one’s own physical or cognitive limitations may hinder an optimal use of the technology adds to the service users’ worries about own safety related to the limitations of the technology. Another of the reablement user in our study (reablement user 1, municipality 3) shares that she experienced falling outside her house and been unable to use the alarm since it only works inside the house. Luckily for her, she was discovered by construction workers who helped her up. Shortly after, the homecare service arrived as well. To our question whether she felt confident to use the alarm in case of an emergency, the user answered that she will be able to use the alarm if she is not dizzy or confused because of the fall.

#### Dementia users

The dementia users in our study report that they often forget to use assistive technology, or they forget that they have it at all. This is the case especially when technological devices such as pendants do not make any sounds unless activated. Other users report putting the assistive devices in drawers to avoid the sound the devices make and then forgetting about having the device. The following quotation from an interview with a service user and their family caregiver shows some of the challenges service users with dementia experience in handling the technology:

I: Whom do you contact in case you feel unwell or need some help?

C: She has the alarm.

U: Yes, I have the alarm.

C: Even though she once fell and hurt herself, she did not release the alarm. But I do not think that the alarm would have helped her anyway.

I: Why wouldn’t it?

C: She did not manage to release the alarm.

I: Is it because you did not reach the alarm?

U: I forgot having the alarm. But I did call my daughter and she came quickly. They were just around the corner, doing groceries, and by the time she came, I have managed to get myself up. Had I only managed to push the button….

C: It would have been better if they did not have to remember releasing the alarm, if falls were registered by some kind of sensors.

(dementia user 1 and family caregiver, municipality 1).

### Theme 3: feel safer with AT when someone attends to the user

The fear of falling and not being discovered is a common source of anxiety among the service users in our study as demonstrated in previous sections. This is especially the case for service users with frail health who have already experienced fractures due to falling and/or other conditions that have required hospitalization. The interview below illustrates such a situation. The reablement user lives with his spouse and despite being provided with a walking stick, walking frame and a pendant alarm, the user is very much dependent on help from his wife:

I: Do you feel you can live an independent life with the help you receive from the municipality?

U: Yes, if I do not fall again.

I: If you do not fall again?

U: If I fall, I would not be able to get up without holding to something. I am that weak.

I: Now that your wife is here and can help you….

U: Yes, she has lifted me several times already.

I: But what if she is not around?

U: I do not know what I would have done without her, she is amazing. [….]

C: You have the alarm, but….

U: Yes, but how much time does it take before they arrive, I do not know.

(reablement user 2, municipality 2)

Some of the service users in our study report that having someone to contact in case of emergency is more important to them than having AT. This is often related to their deteriorating health and weakened ability to use the technology. Service users and caregivers who know whom to contact in case of need, report feeling much safer should an emergency occur, regardless of whether they have the pendant or not.

When asked how safe he feels in his own home, one of the reablement users in our study stated that he needs someone to call to and that the alarm is not really a device that he relies much on:

I: How safe do you feel in your own home?

U: Safe? Well, the thing is that we do not have a person to call to in case of emergency. We do not. We do have the alarm, but I have no idea of how to use it. I know the emergency number. And we have called this number several times with help of the homecare services.

(reablement user 3, municipality 5)

For other informants in our study, having someone who attends to them is what gives them a sense of safety. This is especially the case for older people living alone, as seen in the interview with this reablement user:

U: Well, I told the homecare staff, that I would rather have someone who can assist me with things and teach me things. Or just go out for a walk. And now I need somebody I can support myself to when going to the shop. But I do not have anybody, and I still do the groceries alone and use a walking stick even inside the house. When I go outside, I use two walking sticks. This is what I have been offered. (reablement user 2, municipality 7)

This service user shares that she has been living alone for a very long time. Her husband passed away many years ago and she has no children who can visit her and assist her with practical matters. Loneliness seems to be an issue for this particular user and for other participants in our study, and it is an issue that is not easily dealt with despite the availability of AT.

#### Dementia users

Knowing that someone is attending to the service user, even when the service user is equipped with a number of assistive devices, seems to be of particular importance especially for the family caregivers of home-dwelling older people with dementia. The service user mentioned in the quotation below, is equipped with an electronic medicine dispenser, stove guard, electronic calendar, pendant and a GPS. The family caregiver of this service user has experienced that her mother forgets taking her medication despite being provided with AT to remind her doing so. The family caregiver shares the following:

C: What makes me safe, and I have tried to explain this to my mother, is that when I go to work every day, it is good to know that someone is attending to you [speaks directly to the service user]; that someone checks on you and makes sure that you have taken your medication. It is a bit safer for me to know that someone has visited you.

(dementia user 1 and family caregiver, municipality 2)

In the larger context of promoting independent living in one’s home for as long as possible, the different situations, in which home-dwelling service users with dementia, reablement users and family caregivers of these two service groups feel safe, unsafe or safer when provided with AT may have important implications for the service delivery to these two service groups.

## Discussion

Our findings indicate that even though most of the service users in our study had been provided with a number of different assistive devices, such as walking frames, rollators, medicine dispensers, GPS and others, when asked whether they felt safe continuing living at their homes, the majority of them would refer to the pendant as a source of safety or unsafety. Both the reablement users and home-dwelling older people with dementia felt safe when they knew how to use the alarm. The knowledge of the application of technology was related to the fact that the service users had either tested the alarm or released the alarm and experienced getting help. It seems that the experience of getting in contact with somebody who can provide help gives a feeling of safety for both groups of service users. The family caregivers felt also more at ease knowing that their family members can operate the technology. This finding is in line with existing related research on use of AT. Berge [7] for example, shows that some users experience the intended effects from telecare, such as increased safety; and that it is contextual factors such as the sense of control when living at home or the threat to their safety when living alone, that influenced how people reasoned about the implementation of telecare. However, while Berge [7] does not distinguish between different types of service users, our study shows that both home-dwelling service users with dementia and reablement users may benefit from AT as long as they know how to properly use the technology. A possible policy and service delivery implication of this finding is that providing service users and their family caregivers with timely information about the functioning of AT and ensuring that service users have tested the technology may increase their confidence in using AT, which in turn, may have a positive impact on their sense of safety continuing living at their homes.

Having the knowledge of the use and application of AT is not enough to create a feeling of safety. In fact, for some users, the awareness of the limitations of the technology, sometimes combined with an awareness of own physical and/or cognitive limitations is a source of anxiety, especially when the service user has experienced these limitations. These are situations when the service user has fallen and not been able to contact the call center due to the technology’s limited scope of functioning or due to own physical limitations. For some users even the awareness that this may happen seems to be a source of anxiety and a reason to feel more dependent on their family caregivers.

Furthermore, several of the service users in our study expressed that they would feel much safer if somebody attended to them, and that they needed more human interaction. This finding corroborates findings of other research that describes safety as multidimensional, and in which social as well as emotional and mental safety are linked positively to the availability of home care and developing trusting relationships with healthcare staff [12].

Characteristics associated with ageing such as retirement and loss of the workplace as an arena for physical and social participation, as well as chronic illness and functional limitations, can impose constraints on physical and social engagement [31]. Hence, older people and other impacted groups can be exposed to loneliness and other unfavorable consequences. For some of the informants in our study, especially those with reduced opportunities for social interactions with family and friends, AT, while potentially helpful in maintaining or regaining their functioning, might threaten the few opportunities one has for needed human interaction.

The need for more human interaction despite the availability of AT and despite the users’ having knowledge of the use and functioning of AT shows the limitations of AT and may be seen as an unintended consequence of the living-at-home for as long as possible political ambitions. The need for more human interaction was expressed by both the service users and their family caregivers. Knowing that somebody is attending to the service users seems to be of particular importance especially for the family caregivers of service users diagnosed with dementia. This is also in line with prior research showing that family caregivers worry about the safety of their family members especially diagnosed with dementia [32, 33]. Our findings indicate that it is often the family caregivers who request AT for their family members who suffer from dementia. Even so, they recognize that technology is not enough to keep the user safe in their own home.

To summarize, home-dwelling older adults receiving reablement and dementia care at their homes are two major target groups for assistive technology in Norway. This is related to AT’s promise of enabling older adults to live at home for as long as possible and thus alleviating public expenditure on long-term care. However, the two groups reflect two crucial differences in adaption and use of assistive technology. First, while we can expect that the use of AT will increase over time for the dementia group, the purpose for the users receiving reablement is that the need for and the use of AT will decrease over time. Assistive technology and services serve different purposes for the two groups. For the former group, the aim is to regain functioning, while maintaining functioning is key for the latter. However, what both groups have in common, is the need to feel safe at home.

To even better understand these findings, we draw on perspectives from Actor-Network Theory (ANT) – a theoretical framework within Science and Technology Studies (STS) developed by Latour [34]. Deploying the concept of ‘actant’, we understand assistive technology not as a neutral tool, but rather as an agent that influences the relations between service users and their environments. In ANT, an “actant” is an entity, whether human or not, that plays a role in a network. Actants can be individuals, groups, objects, ideas, technologies, institutions, or any other element that contributes to the formation and functioning of the network. Actants are considered to have agency and be able to influence the network. As seen in the interviews, the assistive technology does play an important role in the everyday lives of the service users and their family caregivers. The aim of the assistive technology is to increase older people’s sense of safety at home. Rather, as we demonstrate in our analysis, technology may have a limiting effect on the everyday life of the service users and their family caregivers. For example, because of the alarm’s limited geographical reach, some of the service users in our study felt anxious and were staying at home, rather than interacting more actively with their environment. Also, the family caregivers of the service users were indirectly restricted by the limitations of the technology as they must plan their own daily routines, such as doing the groceries, in order not to be away from the user for too long. In such cases, technology seems to have power over the service users and their family caregivers, rather than vice-versa. Hence, an unintended consequence of technology if not adapted properly to the specific needs of the user group, may be an increased dependency on the family caregiver and/or sense of unsafey and even anxiety around the use of AT.

### Methodological considerations

It is a strength that the selection of municipalities differ in terms of size, geography, urbanicity etc. This study was part of a larger research project evaluating the Norwegian Government’s Plan for the health care sector, (‘Care Plan 2020’). The use and acceptance of AT among home-dwelling older adults was one of many different topics we inquired into during the interviews with older adults and their family caregivers. Our findings therefore lack the depth and specificity that in-depth interviews on the topic of use and acceptance of assistive technology could have provided.

The municipalities included in this study were sampled from a list of municipalities that took part in projects for developing services for people with dementia living at home and municipalities that have developed the service reablement care. It is therefore possible that the municipalities in our sample have had particularly strong focus on developing primary care services, of which reablement care and dementia care are part of. Further, due to local variations in the provision and implementation of AT in Norwegian municipalities, we caution against drawing strong conclusions from our findings.

Our data was collected between November 2018 and August 2019 and analyzed at a later point in time. We therefore recognize the fact that new types of AT for home-dwelling older adults might have been developed and offered to the study group of individuals that our study focus on.

## Conclusion

For both groups successful use of AT has the potential to significantly reduce overall costs, improve the quality of life for the user and provide a sense of relief for the family caregivers of the users. However, AT alone does not seem to create a sense of safety. Rather, it is the appropriate use of AT within the context of the interactions between service users, their relatives and the healthcare staff that makes people feel safe. Moreover, there are some important differences between the two service groups regarding the progression of their need for AT, the purpose of providing AT (maintaining vs. regaining functioning) and hence need for re-adapting AT to these two service groups. For service users with dementia, AT may help the older person live longer at home thereby postponing institutionalization, given that the user feels confident handling the technology and that technology is timely re-adapted to the user’s changing needs. For reablement users, on the other hand, given that the purpose of providing the user with AT is regaining functioning, providing the right type of technology and then adapting it to the user’s changing needs may be a way of enhancing their safety and recovery. Municipal assistive technology services for older people should therefore be adaptive to differences in needs among different user groups.

### Electronic supplementary material

Below is the link to the electronic supplementary material.

Supplementary Material 1

## Data Availability

No datasets were generated or analysed during the current study.
