# Supplementary material for: Does assistive technology contribute to safety among home-dwelling older adults?
Source: BMC Health Serv Res. 2024 Jun 19;24:750. doi: 10.1186/s12913-024-11185-8 (PMC11188293; doi:10.1186/s12913-024-11185-8)
Supplement: Supplementary file 1 — Supplementary Material 1 [file 12913_2024_11185_MOESM1_ESM.docx]

**APPENDIX**

Below is the complete interview guide that was used in component 3 of the research evaluation of the Care Plan 2020. Component 3 explored the effects of services for home-dwelling older adults with dementia and older people receiving reablement. The interview guide was developed especially for the research evaluation of Care Plan 2020 and is thematically organized. The questions were tailored to explore the experiences that reablement users and dementia users had with the municipal healthcare services they were receiving.

1. The service user's description of the help

Please describe the help/care you receive from previously mentioned people (Note for interviewers: this can be several types of help, e.g. morning or evening care, help with medication, training, shopping for food, cooking, help with cleaning, other practical help around the house, social contact, etc.).

Please describe the help/care you receive from the person who is here with you today (e.g. what you are getting help with).

2. Perception of the care provision

2.1. The user's perception of the care provision

Can you tell me a little about the help you receive from your municipality?

- How long have you received this type of care services from the municipality?

- Who comes to your home (e.g. nursing assistants, nurses, physiotherapists, occupational therapists)?

- What other type of contact do you have with the staff? (e.g. text message, phone call, video visit, visit to the office of the skilled workers)?

- Do you think that the time the healthcare professionals spend on you is sufficient?

- How do they meet you? (degree of patience, respect, kindness, empathy)?

2.2. Relatives' perception of the care provision

Can you tell me a little more about the healthcare professionals from the municipality who provide assistance to [name of the user]?

- Do you have any direct contact with them?

- What type of contact do you have with the healthcare professionals? (e.g. face-to-face, phone call, email, text message, video visit?)

- Do you think that the time they spend on [name of user] is sufficient?

- How do the healthcare professionals meet [name of user]? (degree of patience, respect, empathy, communication)

- How do the healthcare professionals meet you?

- Do you feel that you can trust the staff to provide the help and care that [name of the user] needs (please elaborate)?

3. Coordination

3.1. The user's assessment of the coordination of the services

Now I want you to consider how the staff in the homecare services work together to give you the help you need:

- First, I want to ask what kind of health- and care services you have received in the last two years (for example hospitalization, GP, short-term or rehabilitation place in a nursing home, home rehabilitation, home help and home nursing care)?

- How well do you experience that these services work together? For example, is necessary information about you passed on to those who need it, so that they know about your condition and needs? Have you experienced that you have to repeat your story to the different healthcare professionals?

- Do you receive sufficient information about how the health care professionals view your condition and what help and treatment you need, or do you lack such information?

- Do you know whom to contact (and how to contact them) in case you have questions about your condition or need more help? Can you contact that person any time? If you want to contact one of the staff, how easy or difficult is it to do so?

3.2. Relatives' assessment of the coordination of services

Note to interviews: Ask the same questions to the relative/family caregiver, but with the name of the user as the subject.

4. Perception of patient-centered approach

4.1. User’s perception of patient-centered approach

Now I would like to ask you about how you experience the meetings with the healthcare staff:

- Do you feel that the staff is meeting your needs?

- Did you get to choose the type of help you receive? Do you and the staff have the same view of what you need help with, or do you have different opinions? Are you lacking any help?

- Do you feel that the help you receive enables you to live the life you want?

- Were specific goals set for the help you received (what was to be achieved)? Did you contribute setting up these goals?

- Have you achieved your goals? Have the healthcare professionals assessed the extent to which you have achieved your goals? What has helped/hindered you in achieving your goals? How helpful have the healthcare professionals been in achieving your goals?

4.2. Relatives' perception of patient-centred approach

- Have you been involved in the decisions about the help for [name of user]?

- Do you think that the help [name of user] receives, is contributing to him/her living the life he/she wants and to the best of his/her ability?

5. Preventive care

5.1. The user's perception of preventive care

Now I would like to ask you a few questions about how the services have contributed to you being able to live at home for as long as possible:

- How much and in what manner does the healthcare staff help you to live an independent life?

- Are you sufficiently prepared to take care of yourself in the future?

- Do you know what to do in case your health condition gets worse or in case of an emergency?

- What kind of support do you have in case you feel unwell?

5.2. Relatives' perception of preventive care

- To what extent have the services helped (or not) to maintain [name of user’s] independence?

- What kind of information have you been given about how [name of the user] can continue to live safely and independently at home? Is the information easy to understand? Is it useful? Is there any information you lack?

- To what extent does the healthcare staff contribute to [name of the user] being able to manage their everyday lives?

- Is there anything you can do so that [name of the user] can manage as much as possible by themselves?

- Do you know what to do if [name of the user's] condition worsens or something goes wrong?

6. Safety

6.1. The user's perception of safety

Now I would like to ask you a few questions about how much the staff contributes to you feeling safe and secure at home:

- How safe do you feel at home? (expand and specify)

- Have the healthcare professionals discussed safety measures to help you prevent incidents such as falls or taking a wrong medication?

- Have the healthcare professionals asked you about any side effects related to your prescribed medication?

- Has anyone explained to you what you are supposed to do in case you experience side effects of your prescribed medication?

- Have you been offered improvements of the physical environment of your home to increase your safety? Have you been provided with any assistive devices? If so, what kind of devices and have they contributed to you feeling safer?

6.2. Relatives' perception of safety

Note to interviews: Ask the same question to the relative, with the user as the subject.

7. Quality of the services

7.1. The user's perception of the quality of the services

- Do you think the services you receive from the municipality have improved or worsened in the time you have received them? What has improved/worsened?

- Is there anything you would like to add (e.g. suggestions for improvements)?

7.2. Relatives' perception of the quality of the services

- Do you think the services that [name of user] receives from the municipality have improved or worsened in the time the user has received them? What has become better/worse?

- Is there anything you would like to add (e.g. suggestions for improvements)?
